# Supplementary figures and images for: SON protects nascent transcripts from unproductive degradation by counteracting DIP1
Source: PLoS Genet. 2019 Nov 15;15(11):e1008498. doi: 10.1371/journal.pgen.1008498 (PMC6881055; doi:10.1371/journal.pgen.1008498)

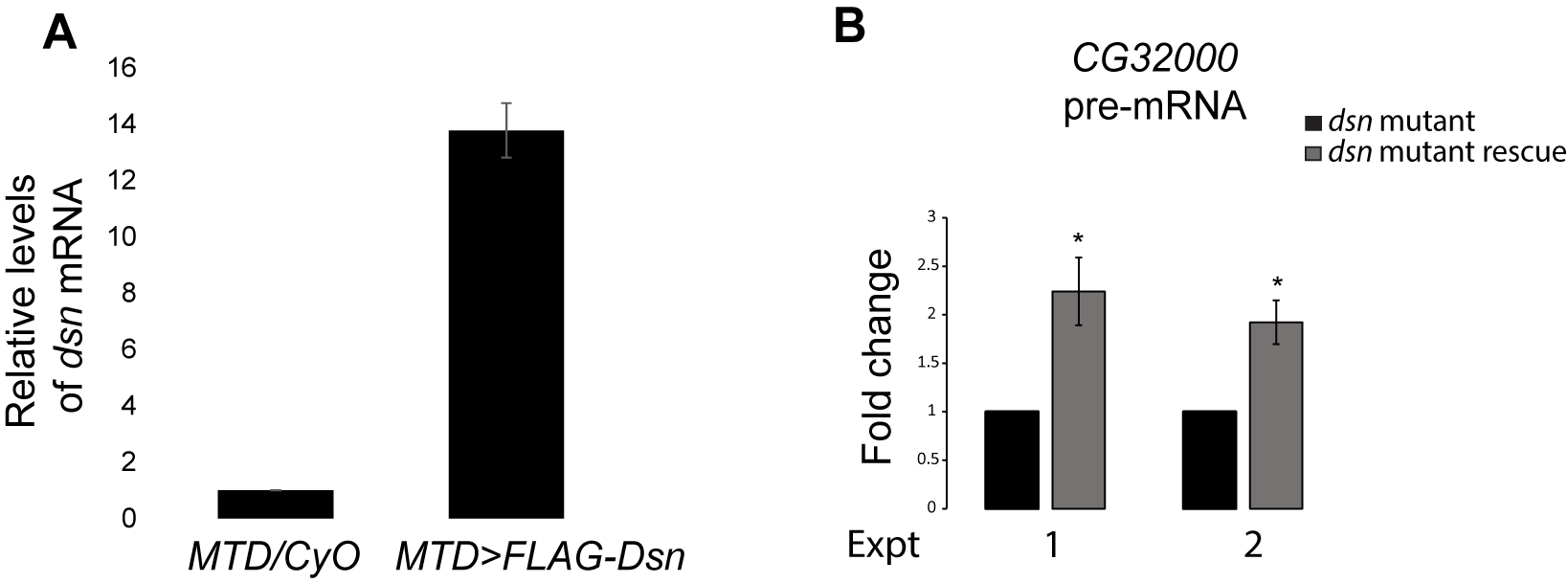

Supplement: S1 Fig — (A) RT-qPCR showing over-expression of dsn mRNA in MTD>FLAG-Dsn versus MTD/CyO ovaries. N = 3 technical replicates. (B) CG32000 pre-mRNA expression was rescued in the dsn mutant rescue ovaries as compared to dsn mutant. A total of 2 independent experiments were done. (TIF) [file pgen.1008498.s001.tif]

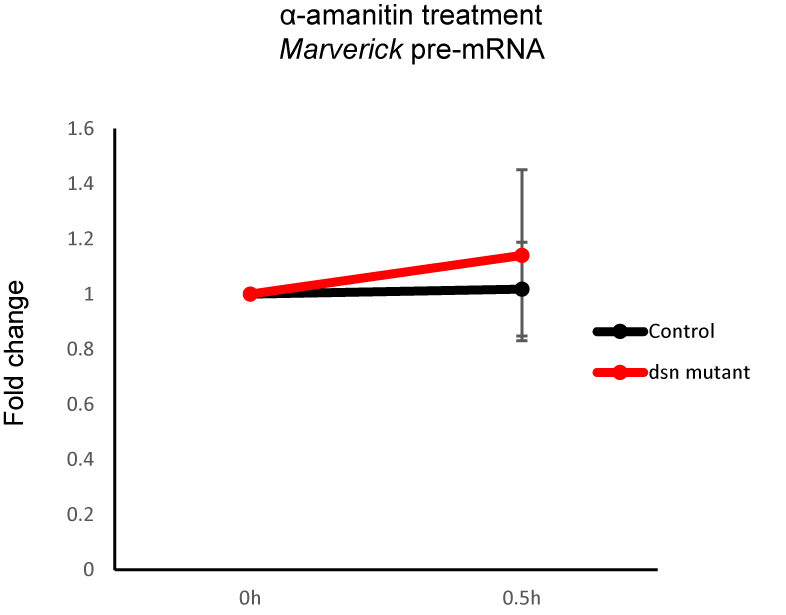

Supplement: S2 Fig — Chart showing the levels of Marverick pre-mRNA in the ovaries of control and dsn mutant flies before and after 0.5h of α-amanitin treatment. Error bars depict SD from three biological replicates. (TIF) [file pgen.1008498.s002.tif]

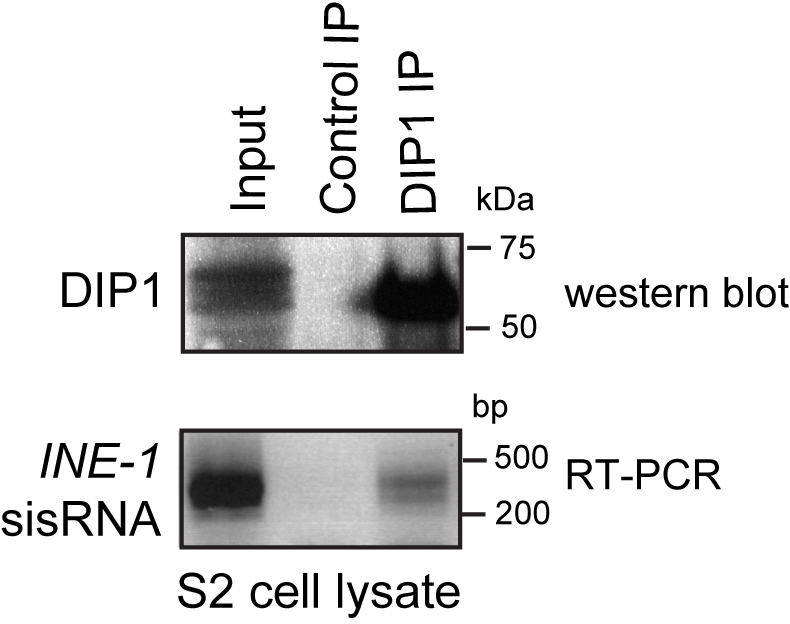

Supplement: S3 Fig — Western blot showing enrichment of DIP1 in DIP1 immunoprecipitate in S2 cells. RT-PCR depicting enrichment of INE-1 sisRNA in DIP1 immunoprecipitate. (TIF) [file pgen.1008498.s003.tif]
