# Supplementary material for: SON protects nascent transcripts from unproductive degradation by counteracting DIP1
Source: PLoS Genet. 2019 Nov 15;15(11):e1008498. doi: 10.1371/journal.pgen.1008498 (PMC6881055; doi:10.1371/journal.pgen.1008498)
Supplement: Attachment — Submitted filename: Response to reviewers.docx [file pgen.1008498.s004.docx]

*Reviewer #1: My remaining concerns have been satisfactorily addressed in this second revision. I agree with the authors that removal of the data with the Dsn antibody (Figure S2, with which I had questions about validity) does not affect the main conclusions of the paper.*

Response: Thank you for your comments!  *Reviewer #2: Here are what would be my major highlights of this work :
- In drosophila ovaries, Dsn localizes to the satellite body where active decay of INE-1-containing introns by DIP1 occurs after splicing.
- Dsn is a negative regulator of DIP1 activity via inhibition of sumoylation and, likely, of subsequent post-translational DIP1 modifications.
- This Dsn-mediated DIP1 inhibition protects the spliced introns less efficiently than the corresponding pre-mRNAs.

I think these data warrant a paper in PLOS Genetics but I would like to warn the reader against some overstatements found in the Abstract and working model (Figure 5):
- I am not convinced that “nascent transcripts are already bound by DIP1 independently of Dsn” (page 9 bottom of first paragraph)*

Response: As mentioned in our response previously, we performed DIP1 immuno-precipitation in control and dsn mutant ovaries, and did not detect any increase in binding of DIP1 to CG32000, CG2316 and Ank pre-mRNAs in dsn mutants (new Figure 3G). This experiment argues that the binding of DIP1 to nascent transcripts is independent of Dsn in the ovaries.

*- There is no evidence either that, page 11 :” Upon splicing, (…) Dsn would be released from the transcripts, “*

Response: Studies done in various other model systems have shown that SON regulates splicing by modulating the splicing regulators and factors (Lu et al., 2013, Nat Cell Biol, Sharma et al., 2011, J Cell Sci, Sharma et al., 2010, Mol Biol Cell, Lu et al., 2014, WIRE RNA). It is also commonly known that splicing regulators disassemble from the transcripts after splicing so that they can be recycled. The relevant citations have been added in the discussion.

*A possible alternative version of the Abstract would be :
Gene expression involves the transcription and splicing of nascent transcripts through the removal of introns. In Drosophila, a double-stranded RNA binding protein Discointeracting protein 1 (DIP1) targets INE-1 stable intronic sequence RNAs (sisRNAs) for degradation after splicing. How nascent transcripts that also contain INE-1 sequences escape degradation remains unknown. Here we observe that these nascent transcripts can also be bound by DIP1 but the Drosophila homolog of SON (Dsn) protects them from unproductive degradation in ovaries. Dsn localizes to the satellite body where active decay of INE-1 sisRNAs by DIP1 occurs. Moreover, the pre-mRNA destabilization caused by Dsn depletion is rescued in a DIP1 heterozygous mutant, suggesting that Dsn is a negative regulator of DIP1. In fact, Dsn is a repressor of DIP1 posttranslational modifications (primarily sumoylation) that are assumed to be required for efficient DIP1 activity. This Dsn-mediated reduction of DIP1 posttranslational modifications is not sufficient to totally abolish DIP1-mediated sisRNAs decay.*

Response: Thank you for the suggestion. We have incorporated most of the suggested changes, and the new abstract reads as follows:

Gene expression involves the transcription and splicing of nascent transcripts through the removal of introns. In *Drosophila*, a double-stranded RNA binding protein Disco-interacting protein 1 (DIP1) targets INE-1 stable intronic sequence RNAs (sisRNAs) for degradation after splicing. How nascent transcripts that also contain INE-1 sequences escape degradation remains unknown. Here we observe that these nascent transcripts can also be bound by DIP1 but the *Drosophila* homolog of SON (Dsn) protects them from unproductive degradation in ovaries. Dsn localizes to the satellite body where active decay of INE-1 sisRNAs by DIP1 occurs. Dsn is a repressor of DIP1 posttranslational modifications (primarily sumoylation) that are assumed to be required for efficient DIP1 activity. Moreover, the pre-mRNA destabilization caused by Dsn depletion is rescued in DIP1 or Sumo heterozygous mutants, suggesting that Dsn is a negative regulator of DIP1. Our results reveal that under normal circumstances nascent transcripts are susceptible to DIP1-mediated degradation, however intronic sequences are protected by Dsn until intron excision has taken place.

*Minor comments:
- Figure 2F: Dsn should also point positively toward INE-1 introns (using a broken arrow ?) to emphasize the fact that the amount of INE-1 is also somewhat reduced in the absence of Dsn (see Figure 2B)*

Response: It is true that INE-1 is also reduced, but that is an indirect effect because of pre-mRNA decay. Thus, we prefer to point the arrow to the most direct effect, which is on the pre-mRNA, to avoid confusing the readers.

*- Since Figure 1D was removed, Figure 3D should be removed too.*

Response: We disagree. Figure 3D has nothing to do with the Dsn antibody. It is a RT-PCR checking transcript levels of dsn.

*- I could not find how Dsn RNAi was performed in ovaries (Figure 3)*

Response: In Figure 4F, it was shown that dsn RNAi in the ovaries was performed by driving RNAi using vasa-Gal4. The genotypes of the fly strains were also provided in the methods. We now also added a section in the methods to describe how dsn RNAi was performed in the S2 cells as follows:

**siRNA-mediated knockdown**

For dsRNA knockdown, experiments were performed as described previously [35]. S2 cells were treated with 15 µg of dsRNA once for four days before cells were harvested. Primers used for generating the template for in vitro transcription were reported previously [16].

*Reviewer #3: This manuscript describes a process by which pre-mRNAs carrying introns that are potential targets for degradation are protected until splicing has occurred. The authors primarily focus on the Drosophila ovary, where they have previously implicated the proteins DIP1 and Dsn (the Drosophila homolog of SON) as regulators of gene expression. They report that a FLAG-tagged version of Dsn localizes to the satellite body (a nuclear body where DIP1 also localizes), and a loss of function mutation in Dsn results in loss of RNA that carries INE-1 elements, which is opposite to the phenotype of loss of function mutations in DIP1, and suggests a protective role for wild type Dsn. They hypothesize that the two proteins act in opposition via Dsn regulation of the RNA-binding DIP1. RNA-IP experiments with a DIP1 antibody support that DIP1 binds to INE-1 RNAs more strongly than to other RNAs, and, in Dsn mutants, DIP1 binding to INE-1 pre-RNAs is unchanged relative to wild type, suggesting that Dsn may regulate DIP1 activity rather than RNA-binding. The authors use western blot analysis to support that DIP1 is sumoylated, likely by Lwr since modified DIP1 is decreased in oocytes heterozygous for an allele of lwr, and that SUMOylation of DIP1 increases in a Dsn mutant background. This leads to a model wherein DIP1 bound to pre-mRNAs carrying INE-1 sequences is held inactive by a SUMOylation-inhibiting activity of Dsn; after splicing, Dsn would no longer inhibit SUMOylation, which would lead to activitation of DIP1 activity and subsequent degradation of INE-1 introns.

The overall story is a very interesting example of RNA biology - how nascent transcripts can be protected when spliced introns are targeted for degradation is likely to be of broad interest to the readership of PLoS Genetics, and the paper addresses some specific mechanistic insights. I am overall positive on the paper and commend the authors on pursuing this interesting biology, and I recognize that the authors have already been through peer review at another journal. However, I believe that several aspects of the manuscript should be addressed before publication. My specific comments follow.

Comments that may require additional evidence/analysis:

1. for the super-resolution deconvolution microscopy, it would be helpful to state the specific type of microscopy in the main text (I believe it is STED). In addition, the analysis of overlap performed in the figure is not described in the main text or methods - are these single optical slices? Were they chosen for any specific reason, or are the “representative”? In addition, some summary of the percent overlap of the two signals overall would be helpful in addition to the examples shown. Finally, I am unclear on the authors’ conclusions from this experiment - they refer to DIP1 and FLAG-Dsn as forming an “intermingling network” - what does this phrase mean?*
Response: Thank you for the very encouraging comments and appreciating our study! We have now described the super-resolution data in greater details as follows:

(page 6) To investigate further, we examined the localizations of DIP1 and Dsn more closely by super-resolution deconvolution (STED) microscopy. Under the super-resolution microscope, the localization patterns of DIP1 and FLAG-Dsn were better resolved. Interestingly, DIP1 and FLAG-Dsn did not overlap completely. Figure 1C shows a representative single optical section of the satellite bodies. Four different regions of the satellite bodies are presented. Measurements of signal intensities showed that DIP1 and FLAG-Dsn only partially overlapped, where they appeared associated closely with each other in a network (Fig. 1c).

*2. in Figure 3C, please use statistical tests to compare binding of DIP1 to INE-1 RNAs relative to the Act5C control RNA. (It is surprising that statistics are used in most other comparisions in the paper, but not here.)*

Response: As mentioned in our previous response to reviewer #2, because of the huge variation between biological replicates, the statistical test will not be significant. The experiments were done on different days and we believe that the variability is due to batch to batch variation. However, it is important to note that within each single experiment, the enrichments of pre-mRNA were always greater than that of actin5C. Hence, the trend that DIP1 binds to pre-mRNAs above background is very clear. We believe that in this context, it is misleading to lump the results from the 3 experiments together and make a conclusion that is based on p-value. *3. the qRT-PCR experiments in figure 2b and c are well controlled with analyses of genes that do not harbor INE-1 elements. The experiments in figure 2d and e are not controlled - the same analysis (at least one) should be done with a gene that does not harbor an INE-1 element (e.g. Myoglianin or Maverick) to show that it the effect is specific to the proposed mechanism.*

Response: We have now added the requested analysis (Supplementary figure 2). The control Marverick pre-mRNA did not show any change in its stability in dsn mutant ovaries.  *4. The authors state that their western exposures are non-saturating, but the 44kDa DIP1 bands in Figure 4d do appear saturated, making comparisons between lanes challenging. Since Figure 4f represents the only experiment tying dsn to their proposed model of DIP1 regulation by SUMOylation, ideally these data would be better presented and more convincing, particularly since at least one trial shows no clear evidence of a change. Similarly, ideally these data would be quantified and compared statistically.*

Response: The main focus of the western blot is really on the upper (modified bands), which do not appear saturated. Furthermore, we used Actin as a loading control under non-saturation conditions. To explain this result more clearly as suggested by the reviewer, we have added in greater details in the text:

(page 10) In two out of three biological replicates, the abundance of the sumoylated forms of DIP1 was up-regulated in *dsn* mutant ovaries (Fig. 4d). We believe that the extent of up-regulation of sumoylation is greater in Expt 2 and 3 than in Expt 1. In Expt 1, the upper-most band in the *dsn* mutant lane was stronger than that in the control lane, suggesting that DIP1 is more heavily modified in the mutants than controls. Thus, the result is still consistent with that of Expt 2 and 3. *5. the final experiment in figure 4f confirms that a loss of a copy of DIP1 suppresses the dsn mutant phenotype on CG32000 pre-mRNA levels (importantly, it also provides a second dsn mutant genetic background to confirm that the phenotype is not specific to the one allele used thus far - the authors may wish to highlight this, as it strengthens their evidence). While this is an important and interesting result, it does not speak to the proposed model of DIP1 regulation by SUMOylation - i.e. there is no evidence that a change in DIP1 SUMOylation regulates its activity. This, to me, is a key experiment to tie the SUMOylation data to the rest of the manuscript. Could the authors, for example, examine INE-1 pre-RNAs in the lwr background where DIP1 sumoylation is significantly reduced? It seems that all of the necessary reagents and techniques are in place for this experiment, and there is a clear prediction. Genetic evidence for regulation via DIP1 SUMOylation would solidify their proposed model; in the absence of this evidence, the data are largely circumstantial.*

Response: This is a really great suggestion! During the course of the review, we had done a genetic interaction test between Dsn and smt3. The new data is now in Figure 4G. The corresponding text reads as follows:

(page 11) To further confirm if the activity of Dsn is due to increase in sumoylation of DIP1, we reduced a copy of *smt3* in the *dsn* homozygous mutant ovaries. Indeed, the level of *CG32000* pre-mRNA was rescued (Figure 4G). Thus, we concluded that Dsn regulates pre-mRNA by counteracting sumoylation of DIP1.

*Comments that can be addressed via changes to the text:

1. the authors state that in their dsn mutants, there is a decrease in INE-1 sisRNAs and mRNAs from genes harboring INE-1 (figures 2b-c). This is based on assessment of three genes, CG32000, CG2316, and ANK. Exonic qRT-PCR shows that ANK levels are not reduced, even though pre-mRNAs for this gene are reduced. The authors should be clear that 2 of 3 genes follow the pattern they describe, and comment on why this might be the case.*

Response: We have amended the text as follows:

(page 7) Although there was a decrease in *ANK* pre-mRNA, we did not observe a similar decrease in *ANK* mRNA in *dsn* mutant ovaries, which may suggest a feedback mechanism regulating *ANK* mRNA.  *2. for Figure 4a, please describe what the “Control IP” is (e.g. in the methods or legend).*

Response: We added the information in the methods “As a control, no antibody was added”. *3. the localization of the satellite body to the region of the 4th chromosome is based on DAPI staining only, where a high density of localized staining is presumed to represent the 4th. Since the chromosome is not specifically identified (e.g. through DNA-FISH or POF staining), it seems that wording like “the presumed 4th” would be more appropriate.*

Response: We have added “presumed”.  *4. I am confused by the presentation of data from S2 cells. The authors first report “an enrichment of INE-1 sisRNAs in the precipitates in S2 cells”, which is presented as a positive control of RNA-IP experiment in oocytes. It is unclear to me how S2 cells are a positive control in this experiment (I presume “precipitates” refers to RNA-IP with DIP1 antibody.) They then report the a Dsn knockdown in S2 cells does not change DIP1 binding to CG32000, nor is there a change in CG32000 pre-mRNA in S2 cells - whereas there was a change in oocytes. Finally, they show no change in DIP1 sumoylation in dsn knockdown cells. It is unclear how the negative data from S2 cells help to explain the relationship between Dsn and DIP1, except that perhaps the relationship is specific to the ovary. Is that the point? If so, perhaps the relevance of this could be commented on in the Discussion.*

Response: We have added a paragraph discussing this point:

(page 13) One interesting observation was that the repressive activity of Dsn on DIP1 was seen in ovaries but not in S2 cells. Knockdown of *dsn* in S2 cells did not lead to a dramatic increase in sumoylation of DIP1. This observation can be explained by the fact that most of the DIP1 protein is already being sumoylated in S2 cells, unlike the case in the ovaries. This difference between S2 cells and ovaries may be due to an intrinsic difference in the activity of sumoylation pathway between these two cell types, thus making the ovaries more sensitive to changes in Dsn activity. *5. While the experiments and phenomenology are quite interesting, I confess that I found this paper very challenging to read and understand. I see from prior reviews that a previous reviewer made similar comments and suggestions for improving the manuscript, which the authors opted not to do. Here are specifics that I hope the authors can improve upon:

-it is confusing that the authors are inconsistent in whether they use SON or Dsn - the title uses SON, but most of the Results refer to Dsn, but then the authors revert to SON again in the Discussion. It would be helpful to stick with one name after telling us about both names.*

Response: We have used SON when we are referring to organisms other than Drosophila, and used Dsn specifically for Drosophila.  *-the introduction is very short, and I did not understand the background information until I had read the whole manuscript. It would be helpful to better explain the background information in the Introduction. Specifically, what it INE-1? and, explain sisRNAs - they are “stable”, but they are degraded? Does the term refer to the pre-mRNAs, the spliced introns, or both, or something else? I don’t understand the name. And, how are satellite bodies “microscopically visible”? by staining? (for what?) or by some other means? Also, it becomes evident later that satellite bodies localize to the 4th chromosome region, it would be helpful to know this in the introduction.*

Response: We have added more explanation in the introduction.

(page 4) Stable intronic sequence RNAs (sisRNAs) are intron-containing transcripts that are relatively more stable than their excised counterparts and those that undergo nonsense-mediated decay [4-9] They have been shown to regulate various biological processes such as germline stem cell maintenance and embryonic development [10,11]. The *Drosophila* chromosome four contains an extremely high abundance of INE-1 sequences in the introns [12]. INE-1 belongs to class of transposable element abundant in *Drosophila* [12-14]. As a result, the fourth chromosome is a region where a high density of INE-1 (sisRNAs) is being produced. Here, a double-stranded RNA binding protein Disco-interacting protein 1 (DIP1) binds and degrades INE-1 sisRNAs [15]. This leads to the formation of microscopically visible DIP1-positive nuclear bodies known as satellite bodies around the fourth chromosomes [15]. DIP1 only degrades INE-1 sisRNAs after splicing as pre-mRNAs containing INE-1 sequences were unaffected in DIP1 mutants [15]. It is not understood how such a target specificity is achieved (Fig. 1a). *-I do not understand the statement “nascent transcripts are already susceptible to degradation by binding to DIP1” in the Introduction. I wonder whether this simply means “nascent transcripts are bound to DIP1”, or is there more to this?*

Response: Amended to “nascent transcripts are bound to DIP1”.  *-the results begin with reference to the gene rga - what is this gene, and how is it relevant to the present study? (is it a sisRNA? a satellite body component? Is it perhaps not relevant?)*

Response: We have now provided the explanation.

(page 5) We previously reported that Dsn regulates germline stem cells (GSCs) by repressing the expression of *regena* (*rga*), which encodes for NOT2 (a component of the CCR4-NOT complex) required for the maintenance of germline stem cells [16-19]. *-in describing the localization of FLAG-Dsn, I appreciate the honesty of the authors, but I believe they do themselves a disservice by explaining that the localization of an overexpressed protein may not reflect the endogenous localization before the actual localization is even discussed. I understand that this was added in response to a previous reviewer, but as written, it undermines the confidence of the reader - if the authors do not believe that the FLAG-Dsn localization is valid, the data should not be in the paper. If they do think it is valid, the language of the paragraph should reflect why they are confident that it is so, even with a potential caveat.*

Response: We have amended the section as follows:

(page 6) Although it should be noted that due to over-expression, the localization of FLAG-Dsn may not precisely reflect that of endogenous Dsn, we believe that the protein reflects it endogenous localization due to its ability to rescue the *dsn* mutant phenotype. *-in the Discussion, the authors state that nascent transcripts are bound by Dsn. I do not see the evidence for this in the current manuscript, a citation for this result should therefore be provided (or please correct my error if the data are in fact included).*

Response: We have cited relevant literature from other species that SON binds to nascent transcripts.

(page 11) SON encodes for an evolutionary conserved protein that binds to nascent transcripts and localizes to nuclear speckles [23-27].
